# Investigating the Multidimensionality of the Work-Related Flow Inventory (WOLF): A Bifactor Exploratory Structural Equation Modeling Framework

**DOI:** 10.3389/fpsyg.2020.00740

**Published:** 2020-05-06

**Authors:** Honglei Gu, Zhonglin Wen, Xitao Fan

**Affiliations:** ^1^Cognition and Human Behavior Key Laboratory of Hunan Province, Department of Psychology, Hunan Normal University, Changsha, China; ^2^Center for Studies of Psychological Application, School of Psychology, South China Normal University, Guangzhou, China; ^3^School of Humanities & Social Science, The Chinese University of Hong Kong (Shenzhen), Shenzhen, China

**Keywords:** work-related flow, WOLF, bifactor model, ESEM, B-ESEM, measurement invariance, nomological validity

## Abstract

This study investigated the factor structure of the Work-Related Flow Inventory (WOLF) through the application of the bifactor exploratory structural equation modeling (B-ESEM) framework. Using a sample of 577 Chinese teachers, we contrasted a series of competing models, including CFA, ESEM, bifactor CFA, and B-ESEM models. The results suggested that the B-ESEM structure with three S-factors (absorption, work enjoyment, and intrinsic work motivation) and one G-factor (global flow) was the best representation of the WOLF ratings. The results also supported the composite reliability and the strict invariance of this measurement structure between male and female groups. Relative to males, female teachers showed a higher level of global work-related flow experience. Finally, the nomological validity of WOLF ratings was supported by the statistical relationships of the WOLF factors with job satisfaction and autonomy.

## Introduction

There has been an increasing interest in the construct of flow over the last forty years. Flow is a state of consciousness where people become totally immersed in an activity, and enjoy it intensely (Csikszentmihalyi, 1997). When a person is engaged in some activity of his or her preference, whether it be leisure (e.g., playing chess), sport (e.g., swimming), work, or study, it is more likely that the individual may experience flow. According to flow theory (Csikszentmihalyi, 1975), flow not only helps individuals to have pleasure and satisfaction in the activity, but also improves their self-efficacy and self-esteem and promotes their self-growth and subjective well-being.

Evidence shows that people may have more experience of flow during work as opposed to during their spare time (e.g., Delle Fave and Massimini, 2003). Bakker (2008) discussed that work-related flow experience could be conceptualized as three aspects: absorption, work enjoyment, and intrinsic work motivation. *Absorption* reflects a person’s concentration on, and immersion in, the work. *Work enjoyment* reflects a person’s happy feeling and positive view with regard to the quality of his work. *Intrinsic work motivation* reflects the tendency that a person does the work for pleasure and satisfaction in the work. Flow at work is most likely to occur when a balance is achieved between the demand of a job and a person’s capacity and adequate organizational resources available for doing the job successfully (Bakker, 2008).

### Psychometric Characteristics and Latent Structure of the WOLF

Despite the existence of many methods (e.g., experience sampling method, questionnaires, neuronal indicators, and psychophysiological measures) and psychometric instruments (e.g., the Swedish Flow Proneness Questionnaire, Ullén et al., 2012; the Flow State Scale, Jackson and Marsh, 1996) developed to assess flow, the work-related flow inventory (WOLF) developed by Bakker (2008) is the most widely administered measure in the work context. By design, the WOLF consists of three dimensions: *absorption* (4 items), *work enjoyment* (4 items), and *intrinsic work motivation* (5 items). As discussed in Csikszentmihalyi (1997), these three dimensions are the important components typically included in research for flow.

Research findings have generally supported the psychometric quality of the WOLF. For example, Bakker (2008) found that the WOLF showed good internal consistency reliability as well as test-retest reliability estimates, in addition to good evidence for its convergent, construct, and predictive validity. Other studies in different cultural settings also provided support for the WOLF’s psychometric quality, such as reliability, predictive validity, and convergent validity, etc. The cultural settings in which such evidence came from included South Africa (Geyser et al., 2015), Norway (Christensen, 2009), Spain (Salanova et al., 2006), Pakistan (Zubair and Kamal, 2015), Italy (Colombo et al., 2013; Zito et al., 2015), and Turkey (Zekioğlu et al., 2017). Similarly, the WOLF has also been shown to have good psychometric characteristics (e.g., reliability and validity) when used in the Chinese cultural context (Zeng, 2013; Chen et al., 2016).

With regard to the latent structure of the WOLF, there are some unresolved issues. Using multiple samples of employees from different occupational groups in Netherlands, Bakker (2008) provided empirical support for the three-factor CFA model consistent with the original design of three components for the measure, over the one-factor model (i.e., only the general factor of flow), and a couple of competing two-factor models. In general, the three-factor structure of the WOLF has also been supported in other cultural settings, such as South Africa (Geyser et al., 2015), Brazil (Freitas et al., 2019), Italy (Colombo et al., 2013; Zito et al., 2015), Turkey (Zekioğlu et al., 2017), and China (Zeng, 2013; Chen et al., 2016). However, most research indicated that this three-factor model typically only showed borderline model fit at best (e.g., Bakker, 2008; Chen et al., 2016), especially with the consideration of the widely used criteria for model fit assessment (Hu and Bentler, 1999).

Furthermore, there was discussion that, of the three constructs proposed for WOLF, two of them – work enjoyment and intrinsic work motivation – overlap conceptually (Llorens et al., 2013; Happell et al., 2015), as enjoyment could already be covered by intrinsic motivation. Operationally, as discussed in Ryan and Deci (2000), self-report of enjoyment is often used for measuring intrinsic motivation. In a sample of Australian workers, Happell et al. (2015) found that the items representing the work enjoyment and intrinsic work motivation dimensions of the WOLF loaded on one dimension, and they argued that the two-factor solution (i.e., absorption and work enjoyment/intrinsic work motivation) should be retained instead of the conventional three-factor structure. Likewise, Bãdoiu and Oprea (2018) showed that the two-factor model had a better fit for the sample data of a Romanian population. In fact, even in the original study (Bakker, 2008), work enjoyment was found to be considerably correlated with intrinsic motivation (e.g., ranging from 0.67 to 0.82), suggesting that these two factors conceptually overlap, which led to poor discriminant validity. Other studies (e.g., Geyser et al., 2015) also discussed these two issues (i.e., borderline model fit and two overlapping constructs).

On the practice side, how the WOLF score(s) is used is also inconsistent. Some researchers used the composited flow score as the measure of global flow (e.g., Fagerlind et al., 2013; Zubair and Kamal, 2015), while some others used three subscale scores to represent the three domain components of flow (e.g., Demerouti et al., 2012). Still others treated the global flow as a latent variable with the three subscale scores as its indicators (Salanova et al., 2006). These studies, however, did not provide any rationale or practical guidelines about why the WOLF score(s) should be used as shown in the respective studies. Ideally, the way in which the WOLF score(s) is used should be grounded in, and supported by, the latent structure of the measure, as how the score(s) of the WOLF is composited should be guided by the latent structure of the measure. When the latent structure of the WOLF is somewhat uncertain, we cannot be sure what scoring mechanism would be the best representation of the underlying structure of the WOLF.

Recent research (e.g., Stenling et al., 2015; Gu et al., 2017b; Tóth-Király et al., 2018) that examined different approaches for modeling the latent structure of some psychological measures indicated that a conventional confirmatory factor analysis approach may often fail to adequately capture the more complicated multidimensionality of the latent structure of some measures; more sophisticated modeling approaches may be needed to better model the multidimensionality of some measures. It is likely that we may develop a better understanding about the issues concerning the latent structure of the WOLF as discussed above by considering some more sophisticated modeling approaches that may better capture measurement multidimensionality. With all these considerations, it became necessary to revisit the issue of the latent structure of the WOLF, to develop a better understanding of the multiple issues discussed above.

### Approaches for Modeling Multidimensionality

#### Confirmatory Factor Analysis (CFA)

The confirmatory factor analysis (CFA) is the most commonly used approach to model construct-relevant multidimensionality. CFA, however, is often criticized for its overly restrictive independent cluster model (ICM) assumption, which requires that each item is defined by one, and only one, content domain. This assumption is operationalized in a CFA (ICM-CFA) model by constraining all cross-loadings to zeros, which could lead to unintended consequences, such as inflated factor correlations, poor goodness-of-fit indices, and poor discriminant validity, etc. Indeed, research indicated that the ICM-CFA model, even when the model fit was satisfactory, could lead to inflated factor correlations (e.g., Morin et al., 2017).

#### Bifactor CFA

Bifactor CFA model assumes: (a) the existence of a general factor that accounts for the shared communality by all the items; and (b) the existence of several group factors, which contribute to a common variance shared within each cluster of items, beyond that of the general factor (Reise, 2012; Gu et al., 2015, 2017a). For model identification, orthogonality is assumed between the general factor and the specific factors. Such a bifactor CFA model better represents the multidimensionality of the underlying factor structure because of the coexistence of a general construct (e.g., flow at work) and some specific constructs (e.g., absorption, work enjoyment, and intrinsic work motivation).

#### Exploratory Structural Equation Modeling (ESEM)

Exploratory structural equation modeling provides an overarching framework which integrates CFA and exploratory factor analysis (EFA) into a single structural equation modeling (SEM) model. This model is more appropriate for investigating possible multidimensionality of a measure due to the associations between non-target constructs and imperfect items (Asparouhov and Muthén, 2009). ESEM relies on target rotation, which is a confirmatory form of rotation, to freely estimate cross-loadings. Compared with CFA, ESEM provides more accurate, typically lower, estimates of factor correlations, and these more accurate estimates of factor correlations result in better discriminant validity (Asparouhov and Muthén, 2009; Morin et al., 2016).

#### Bifactor Exploratory Structural Equation Modeling (B-ESEM)

Bifactor exploratory structural equation modeling was recently proposed by Morin et al. (2016) to examine the issue of construct-relevant multidimensionality. B-ESEM integrates both bifactor model and ESEM model into a single analytical framework. This new modeling approach not only allows the coexistence of the general construct and its subdomains (e.g., global flow, and absorption, work enjoyment, and intrinsic work motivation as specific components), but also takes the relations of non-target constructs and items into account. Theoretically, the B-ESEM is the most comprehensive and flexible model that can more accurately describe the complex psychological characteristics.

Compared with B-ESEM, ESEM ignores the possible presence of hierarchically higher order construct(s) (e.g., global flow at work), which can lead to inflated cross-loadings. By contrast, bifactor model, which is essentially a CFA model, neglects the possibility that items may have cross-loadings on the non-target specific factors. The consequence of fixing such cross-loadings to zero is to inflate the variance of the general factor (Morin et al., 2017; Sánchez-Oliva et al., 2017). B-ESEM, theoretically, overcomes these shortcomings as described above.

### Nomological Validity of the WOLF

The nomological validity of WOLF could be supported by appropriate statistical relationships between work flow and external criterion variables such as autonomy and job satisfaction. As Morgeson et al. (2005) discussed, job autonomy reflects how much a job allows an employee to have discretion, freedom, and independence for work scheduling, or allows employees to make the necessary decisions to get the job done. Job satisfaction, on the other hand, is a person’s agreeable or positive emotional state that is based on personal evaluation of one’s occupation or job experiences (Locke, 1976). As Hackman and Oldham (1980) described in their job characteristics model, five important job characteristics (namely, task significance, skill variety, autonomy, feedback, and task identity) generate and enhance a person’s flow experience. Of these five, autonomy seems to have the most beneficial effect on flow (Bakker, 2008; Mäkikangas et al., 2010; Lin and Joe, 2012). Empirical evidence also suggested that autonomy was significantly and positively associated with flow experience. For example, Fullagar and Kelloway (2009) revealed that autonomy was a significantly positive predictor for flow. In addition, many other studies showed that job satisfaction was closely related to work flow or its specific components (e.g., Maeran and Cangiano, 2013; Geyser et al., 2015; Zito et al., 2015).

### The Present Study

We conducted this study with three specific aims. First, we intended to investigate WOLF’s latent structure, by using both conventional and more recent modeling approaches, such as ESEM model, bifactor model, and B-ESEM model, for the purpose of resolving some issues related to WOLF’s latent structure. Second, we intended to examine how invariant the WOLF structure was across gender groups. For this purpose, a series of progressively more stringent invariance conditions (e.g., ranging from configural, weak, strong, and to strict invariance) would be tested. Third, we intended to examine the nomological validity of the WOLF in relation to the relevant constructs of autonomy and job satisfaction, as suggested by the best model that emerged from the modeling analyses under the first aim.

## Materials and Methods

### Participants

The participants were 577 teachers recruited in Zhengzhou, a metropolitan area in central China. The sample’s average age was 36.80 years old (*SD* = 9.04), and their average work seniority was 12.20 years (*SD* = 9.95). The majority of participants were female (71.9%) and married (83.5%). Among the participating teachers, 21.0% were teaching in kindergartens, 40.0% in primary schools, and 39.0% in secondary schools.

### Measures

#### Flow at Work

The *Work-Related Flow Inventory* (WOLF; Bakker, 2008) was used to measure *flow at work*. This measure had 13 items designed to assess three dimensions of flow experience: (a) *absorption* (four items; sample item: “I get carried away by my work”), (b) *work enjoyment* (four items; sample item: “I do my work with a lot of enjoyment”), and (c) *intrinsic work motivation* (five items; sample item: “I find that I also want to work in my free time”). The items had the response scale with 7-points ranging from 1 (never) to 7 (always). For using this measure in the sample of Chinese teachers, the standard procedure of translation and back-translation (Brislin, 1986) was used to translate the original WOLF into Chinese, and both the English and Chinese items of the WOLF were available in Supplementary Table S1 of Supplementary Appendix. Cronbach’s α was 0.92 for the total scale, and 0.85, 0.91, and 0.83 for the three subscales of *absorption*, *work enjoyment*, and *intrinsic work motivation*, respectively. The model-based reliability (i.e., omega coefficient, ω; Sijtsma, 2009) would be estimated and reported in section “Results.”

#### Job Satisfaction

The *Job Satisfaction Scale* (Schriesheim and Tsui, 1980) was used to measure *job satisfaction*. The self-report scale contained six items, with each being rated on a 5-point Likert scale (1 = strongly disagree; 5 = strongly agree). A sample item is, “How satisfied are you with the nature of the work you perform?” Cronbach’s α and the omega coefficient (ω) in the study sample were both 0.86.

#### Autonomy

*Autonomy* was measured by using the subscale of self-determination under the *Psychological Empowerment Scale* (Spreitzer, 1995). The subscale consisted of three items (e.g., “I have significant autonomy in determining how I do my job”). Participants responded to each item on a 5-point Likert scale (1 = strongly disagree; 5 = strongly agree). In this study, Cronbach’s α and the omega coefficient (ω) were both 0.83.

### Statistical Analysis

To achieve the aims of the study, statistical analyses were carried out in three phases. In the first phase of analyses, for understanding the measurement structure of the WOLF, a series of nine alternative models were examined to assess their respective goodness-of-fit, as follows.

Model of unitary dimension:

Model 1: One-factor CFA model (global flow).

Models with two sub-domains*:*

Model 2: Two-factor CFA model (absorption, work enjoyment/motivation).
Model 3: ESEM model (including absorption and work enjoyment/motivation).
Model 4: Bifactor CFA model (B-CFA) with two specific domains (absorption and work enjoyment/motivation).
Model 5: B-ESEM model, including two S-factors (absorption, work enjoyment/motivation), and one G-factor (global flow).

Models with three sub-domains:

Model 6: Three-factor CFA model (absorption, work enjoyment, and intrinsic work motivation).
Model 7: ESEM model (including absorption, work enjoyment, and intrinsic work motivation).
Model 8: Bifactor CFA model (B-CFA) with three specific domains (absorption, work enjoyment, and intrinsic work motivation).
Model 9: B-ESEM model, including three S-factors (absorption, work enjoyment, and intrinsic work motivation), and one G-factor (global flow).

Among the nine models above, Model 1 was the baseline model, which assumed one general factor of global flow without considerations for any sub-domains. Model 2 to Model 5 shared the general assumption of two sub-domains of work flow. Model 6 to Model 9 shared the general assumption of three sub-domains of work flow.

In the first-order CFA models (Model 2 and Model 6), each item was specified to load on the factor (i.e., the content domain) that the item was assumed to measure, and without cross-loadings on any other factors. In the first-order ESEM models (Model 3 and Model 7), all cross-loadings were specified to be freely estimated through oblique target rotation. The B-CFA models (Model 4 and Model 8) assumed that each item simultaneously loaded onto a global flow construct and one of the specific domains of flow, and that all factors were orthogonal (i.e., uncorrelated with each other). As for the B-ESEM models (Model 5 and Model 9), an item was not only defined by the G-factor and by a S-factor of its own, but it also reflected other conceptually adjacent subdomains (i.e., cross-loadings) through orthogonal bifactor-target rotation.

In the second phase of analyses, for the purpose of testing measurement invariance across gender groups, the best fitting model that emerged from the first phase of modeling analyses (i.e., Model 1 to Model 9; described above) was used, and measurement invariance analyses were conducted by using the sequence described in the literature (Millsap, 2011). The analyses tested progressively more stringent invariance assumptions: (a) configural invariance (invariance of factor structure), (b) weak invariance (#a satisfied, plus invariance of factor loadings), (c) strong invariance (#b satisfied, plus invariance of item intercepts), (d) strict invariance (#c satisfied, plus invariance of item uniquenesses), (e) latent variance-covariance invariance (#d satisfied, plus invariance of latent variance-covariance), and (f) latent means invariance (#e satisfied, plus invariance of latent factor means).

In the third phase of analyses, latent factors representing job satisfaction and autonomy were integrated to the retained measurement model to examine the nomological validity of the WOLF.

All modeling analyses were carried out by using the statistical modeling software M*plus* 7.0 (Muthén and Muthén, 2012). In the modeling analyses, the robust maximum likelihood (MLR) estimation method was used, which provides estimates of standard errors and fit indexes appropriate for conditions such as ordinal Likert-scale item responses and data non-normality. For model fit assessment, we considered the following model-fit indices: the Comparative Fit Index (CFI), the Tucker-Lewis Index (TLI), and the root mean square error of approximation (RMSEA) with its confidence intervals (CI). As suggested in the literature (Hu and Bentler, 1999; Marsh et al., 2004), adequate and excellent model fit may be indicated by values greater than 0.90 and 0.95, respectively, on CFI and TLI, and by values lower than 0.08 and 0.06, respectively, on RMSEA. For testing alternative models, as discussed in Chen (2007), ΔCFI and ΔTLI ≥ 0.01 and ΔRMSEA ≥ 0.015 could be considered to suggest a more restrictive model.

## Results

### Descriptive Statistics

Table 1 presents the means, standard deviations, and Pearson correlations for the measured variables. As expected, the three components of the WOLF (absorption, work enjoyment, and intrinsic work motivation) were positively related to each other (*r* = 0.66–0.72, *p* < 0.001). Absorption, work enjoyment, and intrinsic motivation correlated with both autonomy and job satisfaction (*r* = 0.44–0.68, *p* < 0.001).

**TABLE 1:** Means, standard deviations, and correlations of the study variables.

	**1**	**2**	**3**	**4**	**5**
1. Absorption	–				
2. Work enjoyment	0.66***	–			
3. Intrinsic work motivation	0.66***	0.72***	–		
4. Job satisfaction	0.44***	0.68***	0.62***	–	
5. Autonomy	0.49***	0.61***	0.60***	0.75***	–
*M*	4.99	5.05	5.06	3.59	3.68
*SD*	1.26	1.37	1.13	0.82	0.59

*n* = 577. ***p < 0.001.

### Latent Structure of the WOLF

Results of model fit assessment for the nine alternative models, which represented different latent measurement structures of the WOLF as discussed previously, are displayed in the upper portion of Table 2. Based on comparison of the alternative two-subdomain (Models 2 to 5) and three-subdomain (Models 6 to 9) solutions, it is apparent that the three-subdomain solutions had a much better fit to the data than the two-subdomain counterparts. The parameter estimates for the two-subdomain models, which were reported in Supplementary Tables S2, S3 of Supplementary Appendix, further supported the three-subdomain solutions.

**TABLE 2:** Model fit statistics of alternative measurement models (upper) and measurement invariance tests of B-ESEM model (lower).

Model comparison analysis	χ^2^ (*df*)	RMSEA (90%CI)	CFI	TLI					
Model 1: One-factor CFA	553.60 (65)	0.14 (0.13, 0.15)	0.79	0.74					
Model 2: Two-factor CFA	379.57 (64)	0.11 (0.10, 0.12)	0.86	0.83					
Model 3: Two-factor ESEM	503.70 (53)	0.12 (0.11, 0.13)	0.86	0.80					
Model 4: B-CFA: Two S-factors	239.81 (52)	0.09 (0.08, 0.10)	0.92	0.88					
Model 5: B-ESEM: Two S-factors	223.97 (42)	0.09 (0.08, 0.10)	0.94	0.89					
Model 6: Three-factor CFA	325.16 (62)	0.11 (0.10, 0.11)	0.88	0.85					
Model 7: Three-factor ESEM	223.97 (42)	0.09 (0.08, 0.10)	0.94	0.89					
Model 8: B-CFA: Three S-factors	245.15 (52)	0.09 (0.08, 0.10)	0.92	0.88					
Model 9: B-ESEM: Three S-factors	59.99 (32)	0.05 (0.04, 0.06)	0.99	0.97					

**Measurement invariance analysis**	**χ^2^ (*df*)**	**RMSEA (90%CI)**	**CFI**	**TLI**	**CM**	**Δχ^2^ (Δ*df*)**	**ΔRMSEA**	**ΔCFI**	**ΔTLI**

Model A: Configural IN	116.98 (64)	0.05 (0.04, 0.07)	0.98	0.96		–	–	–	–
Model B: Weak IN	163.27 (100)	0.05 (0.03, 0.06)	0.98	0.97	Model A	46.29 (36)	0.00	0.00	0.01
Model C: Strong IN	172.26 (109)	0.05 (0.03, 0.06)	0.98	0.97	Model B	8.98 (9)	0.00	0.00	0.00
Model D: Strict IN	186.04 (122)	0.04 (0.03, 0.06)	0.98	0.98	Model C	13.78 (13)	–0.01	0.00	0.01
Model E: Latent v/c IN	246.62 (132)	0.06 (0.04, 0.07)	0.97	0.96	Model D	60.58 (10)	0.02	–0.01	–0.02
Model F: Latent means IN	273.91 (136)	0.06 (0.05, 0.07)	0.96	0.95	Model D	87.87 (14)	0.02	–0.02	–0.03

*χ^2^ = robust chi-square test of exact fit; *df* = degree of freedom; RMSEA = root mean square error of approximation; CFI = comparative fit index; TLI = Tucker-Lewis Index; CM = comparison model; B-CFA = bifactor CFA; IN = invariant; v/c = latent variance and covariance.*

With the superiority of the three-subdomain solutions clearly supported, we shifted our focus to the comparisons of different forms of three-subdomain solutions (i.e., comparisons among Models 6 to 9). As discussed in Morin et al. (2016), we first compared the CFA (Model 6) and ESEM (Model 7) models, and it was revealed that the ESEM model (Model 7) showed better model fit (ΔTLI = 0.04, ΔCFI = 0.06, and ΔRMSEA = –0.02) than the CFA model (Model 6).

Tables 3 and 4 present the standardized factor loadings and factor correlations of these two models, which also provided support for the ESEM solution. More specifically, most of the target loadings were statistically significant and practically acceptable in the three-factor CFA (|λ| = 0.44–0.91; *M* = 0.74) and ESEM (|λ| = 0.10–0.91; *M* = 0.58) models. CFA factor correlation estimates (*r* = 0.73–0.88; *M* = 0.81) were overall larger than the corresponding ESEM model factor correlations (*r* = 0.18–0.68; *M* = 0.39), which indicated that ESEM results showed better differentiation among the factors. More noticeably, the correlation of work enjoyment with intrinsic work motivation was reduced from 0.88 in the CFA model to 0.30 in the ESEM model. The findings based on this initial evaluation indicated that the ESEM should be the preferred model.

**TABLE 3:** Standardized parameter estimates for three-factor CFA (Model 6) and three-factor ESEM (Model 7) models.

	Three-factor CFA		Three-factor ESEM			
		
	λ	δ	AB (λ)	WE (λ)	IWM (λ)	δ
***Absorption* (AB)**						
*a*1	0.58	0.66	**0.47**	0.05	0.26	0.63
*a*2	0.79	0.38	**0.70**	0.22	*–0.23*	0.28
*a*3	0.83	0.32	**0.87**	*–0.10*	*0.18*	0.27
*a*4	0.87	0.25	**0.91**	*–0.07*	*0.05*	0.24
***Work enjoyment* (WE)**						
*w*1	0.86	0.26	*–0.09*	**0.87**	0.20	0.21
*w*2	0.91	0.17	0.12	**0.87**	*–0*.13	0.13
*w*3	0.86	0.26	*–0.07*	**0.82**	0.30	0.17
*w*4	0.88	0.23	0.16	**0.78**	*–0.07*	0.22
***Intrinsic work motivation* (IWM)**						
*i*1	0.68	0.54	0.23	0.34	***0.33***	0.52
*i*2	0.47	0.78	0.36	0.21	***–0.19***	0.74
*i*3	0.44	0.81	0.41	–0.06	**0.30**	0.74
*i*4	0.63	0.60	*0.07*	*0.40*	**0.38**	0.56
*i*5	0.82	0.32	0.37	0.51	***–0.10***	0.37

*Non-significant loadings (*p* > 0.05) are italicized. Target loadings of the ESEM model are shown in bold.*

**TABLE 4:** Inter-factor correlations for three-factor CFA (Model 6) and three-factor ESEM (Model 7) Solutions.

		Work	Intrinsic Work
	Absorption	Enjoyment	Motivation
Absorption	–	0.73***	0.81***
Work enjoyment	0.68***	–	0.88***
Intrinsic work motivation	0.18**	0.30***	–

ICM-CFA correlations are displayed above the diagonal and ESEM correlations are displayed below the diagonal. **p < 0.01; ***p < 0.001.

Morin et al. (2016) made the suggestion that the second comparison would compare the retained model from the CFA vs. ESEM comparison above with its bifactor counterparts (either B-CFA or B-ESEM). Here, as the ESEM model was retained, we now compare ESEM and B-ESEM models. Although most cross-loadings in ESEM remained small (|λ| = 0.05–0.51; *M* = 0.20), some were large enough to indicate the possibility of an unmodelled G-factor. Out of 26 cross-loadings, seven were between 0.20 and 0.30 (Items a1, a2, w1, w3, i1, and i2), and six were over 0.30 (Items i1, i2, i3, i4, and i5).

As shown in Table 2, the B-ESEM solution (Model 9) had excellent model fit to the data as shown by all model fit indices (TLI = 0.97, CFI = 0.99, RMSEA = 0.05), which substantially exceeded (ΔTLI = 0.08, ΔCFI = 0.05, ΔRMSEA = –0.04) the model fit of the ESEM solution (Model 7). More importantly, the B-ESEM model’s factor loadings reported in Table 5 were indicative of a G-factor, as shown by the substantial and strong loadings from all the indicators (|λ| = 0.40–0.86; *M* = 0.66). Beyond the global flow factor, the loadings on the target-specific factors (|λ| = 0.01–0.59; *M* = 0.37) were substantially larger than the non-target loadings (|λ| = 0.00–0.22; *M* = 0.09). The specific factors of absorption (|λ| = 0.21–0.59; *M* = 0.42) and work enjoyment (|λ| = 0.30 –0.59; *M* = 0.45) were well-defined with generally moderate to large target loadings. Loadings on the intrinsic work motivation S-factor (|λ| = 0.01–0.53; *M* = 0.27) were lower in general than the loadings on the other group factors. In addition, two of five target loadings (Item i2, |λ| = 0.10; Item i5, |λ| = 0.01) on the intrinsic work motivation S-factor were statistically non-significant. These suggested that the intrinsic work motivation S-factor is less well-defined than the other two, but acceptable.

**TABLE 5:** Standardized factor loadings for B-ESEM model with three S-factors and one G-factor (Model 9).

	GWF (λ)	S-AB (λ)	S-WE (λ)	S-IWM (λ)	δ
***Absorption* (AB)**					
*a*1	0.46	**0.34**	0.13	0.16	0.63
*a*2	0.80	**0.21**	–0.11	–0.20	0.27
*a*3	0.66	**0.59**	*0.03*	0.06	0.22
*a*4	0.70	**0.52**	*–0.05*	*–0.05*	0.23
ω		**0.67**			
***Work Enjoyment* (WE)**					
*w*1	0.68	*0.02*	**0.59**	0.13	0.17
*w*2	0.83	*0.01*	**0.40**	–0.15	0.12
*w*3	0.70	*0.00*	**0.52**	0.22	0.20
*w*4	0.83	*–0.02*	**0.30**	*–0.05*	0.23
ω			**0.82**		
***Intrinsic Work Motivation* (IWM)**					
*i*1	0.61	*0.05*	0.14	**0.37**	0.47
*i*2	0.52	*0.00*	–0.14	***–0.10***	0.70
*i*3	0.40	0.18	*–0.06*	**0.33**	0.70
*i*4	0.57	–0.10	0.13	**0.53**	0.37
*i*5	0.86	–0.07	*–0.03*	***0.01***	0.26
ω	**0.94**			**0.42**	

*GWF = general work-related flow. Non-significant loadings (*p* > 0.05) are italicized. Target loadings on specific factors of the B-ESEM model are shown in bold.*

More importantly, the B-ESEM model’s cross-loadings (|λ| = 0.00–0.22; *M* = 0.09) were substantially lower than those of the ESEM model (|λ| = 0.05–0.51; *M* = 0.20). Furthermore, in the B-ESEM solution, only two cross-loadings were between 0.20 and 0.30 (Items a2 and w3), and none were over 0.30. All these findings provided strong support for retaining the B-ESEM model as the best representation of the structure of the WOLF.

For further assessing the appropriateness of the B-ESEM model, we calculated model-based coefficients of composite reliability (Perreira et al., 2018) for both the G-factor and the S-factor, based on the standardized model estimates. The composite reliability of both the general flow factor (ω = 0.94) and the work enjoyment S-factor (ω = 0.82) were very good. The composite reliability of the S-factor for absorption (ω = 0.67) was adequate, and that of the intrinsic work motivation S-factor (ω = 0.42) was marginal.

### Assessment for Gender Group Measurement Invariance

The measurement invariance across gender for the B-ESEM model was assessed, and the findings were displayed in the lower portion of Table 2. The configural invariance model (Model A) showed very good model fit to the data (TLI = 0.96, CFI = 0.98, RMSEA = 0.05). Progressively more stringent invariance constraints were then successively imposed on factor loadings (Model B: weak invariance), item intercepts (Model C: strong invariance), and item uniquenesses (Model D: strict invariance). None of these more stringent invariance conditions caused model fit deterioration beyond the general guidelines (i.e. ΔCFI and ΔTLI ≥ 0.01 and ΔRMSEA ≥ 0.015). However, the model for the invariance of latent variance-covariance (Model E; ΔTLI = –0.02, ΔCFI = –0.01, ΔRMSEA = 0.02) and the model for latent means invariance (Model F; ΔTLI = –0.03, ΔCFI = –0.02, ΔRMSEA = 0.02) were not supported by the data. Further analysis showed that when males’ factor means were fixed to zero for model identification purpose, female teachers’ factor means were statistically higher on the work flow G-factor (*M* = 0.46, *p* < 0.001), but not statistically different on the S-factors (*p* > 0.05).

### Nomological Validity

For the purpose of examining the nomological validity of the WOLF, external CFA factors for job satisfaction and autonomy were included into the B-ESEM model (Model 9), and this expanded model showed very good model fit (χ^2^ = 282.10, *df* = 167, RMSEA = 0.04, RMSEA 90%CI = [0.04, 0.05], TLI = 0.96, CFI = 0.97). As displayed in Table 6, the flow G-factor and two S-factors (i.e. work enjoyment and intrinsic work motivation) were significantly and positively associated with job satisfaction and autonomy. By contrast, the absorption S-factor was not significantly associated with these two external factors.

**TABLE 6:** Correlations between WOLF factors and two external factors (job satisfaction and autonomy) based on the B-ESEM model with three S-factors and one G-factor.

	Job satisfaction	Autonomy
General work-related Flow	0.66***	0.49***
Absorption	–0.09	0.03
Work Enjoyment	0.33***	0.32***
Intrinsic work motivation	0.44***	0.30***

***p < 0.001.

## Discussion

This study is the first attempt to investigate the latent structure of the WOLF by using both CFA and ESEM approaches. Consistent with previous research (e.g., Bakker, 2008; Christensen, 2009; Happell et al., 2015), this study found that the one-factor CFA solution was far from being acceptable, indicating that work-related flow should be considered as consisting of multiple dimensions, rather than of a unitary dimension.

The WOLF was originally designed to assess three inter-related content domains (Bakker, 2008), and the three-factor structure was shown in various samples and in different cultures. However, Rodríguez-Sánchez et al. (2011) and Llorens et al. (2013) discussed that only enjoyment and absorption were the essence of the work-related flow experience. Enjoyment could be considered as some kind of motivation (Davis et al., 1992), and intrinsic motivation might be an antecedent, instead of a core component, of work-related flow (Deci and Ryan, 1985; Llorens et al., 2013). The study by Happell et al. (2015) in an Australian sample showed that the items for two domains, work enjoyment and intrinsic work motivation, loaded onto one dimension, providing support for the argument described above. For the purpose of understanding whether these two components might be combined into one factor, we compared the two-factor CFA (absorption and work enjoyment/motivation) and the three-factor CFA (absorption, work enjoyment, intrinsic work motivation) solutions, and found that the goodness-of-fit of the latter model substantially exceeded that of the former. More importantly, we found that the correlation between work enjoyment and intrinsic work motivation in the three-factor CFA model was indeed high (i.e., 0.88), which was in line with previous findings (e.g., Bakker, 2008; Geyser et al., 2015; Zito et al., 2015). With such findings based primarily on conventional CFA approaches, it is difficult to decide which model should be preferable for WOLF. Therefore, new modeling approaches (e.g., B-CFA, ESEM, and B-ESEM) could be needed to further examine the dimensionality of the WOLF structure.

In line with prior research on multidimensional data, the comparison between the ICM-CFA model and ESEM model in this study revealed that the ESEM model was preferable, as ESEM had better model fit, and the factors showed better differentiations between each other as indicated by the lower inter-factor correlations. The ESEM solution, similar to the three-factor CFA, only considered the subdomains as separate factors for absorption, work enjoyment, and intrinsic work motivation, without the consideration for a possible overarching global factor. The observation of multiple cross-loadings of sizable magnitude (|λ| > 0.20, or even 0.30) in the ESEM model suggested that a global work-related flow factor might be present in the data. The comparison of ESEM and B-ESEM solutions provided support for this possibility. First, B-ESEM had substantially better model fit to the data. Second, the general flow dimension in B-ESEM appeared to be well defined, with the items showing moderate to large loadings on this general flow factor. Third, the composite reliability of the flow G-factor (ω = 0.94) was excellent. Fourth, the specific factors of absorption and work enjoyment were well-defined, while the specific factor of intrinsic work motivation was less well-defined, but generally acceptable. Finally, cross-loadings in the B-ESEM solution were generally lower than those of the ESEM solution.

In general, if the composite reliability (ω) of a specific factor is sufficiently high (e.g., >0.5), it indicates that the subscale score accounts for a meaningful amount of variance beyond the G-factor (Perreira et al., 2018). The findings in this study showed that the specific factors of absorption (ω = 0.67) and work enjoyment (ω = 0.82) had a substantial amount of specificity of its own, over and above the global flow. On the other hand, the specific factor of intrinsic work motivation (ω = 0.42) was less well-defined and had relatively low composite reliability. But three of the five target loadings exceeded 0.3, indicating that this specific factor still had an acceptable degree of specificity beyond the G-factor. Therefore, it is suggested to report the total score and subscale scores of absorption and work enjoyment when using the WOLF in practice. The use of subscale score of intrinsic work motivation should be treated with caution.

As shown earlier, the B-ESEM solution showed the best model fit. In the B-ESEM model, however, Item w3 (“I feel happy during my work”) not only reflected the global work-related flow and the subdomain of work enjoyment, but it also had a substantial cross-loading (λ = 0.22) on the non-target intrinsic work motivation S-factor. This, however, was reasonable, because employees who are happy at work are usually motivated intrinsically by their work (Geyser et al., 2015). Psychometrically, it may not be realistic to require that each item reflects one, and only one, content domain of multidimensional constructs (Asparouhov and Muthén, 2009).

In addition, the findings also provided support for strict measurement invariance of the B-ESEM solution across gender groups, suggesting that this model was well-replicated across subsamples of male and female teachers. For the latent mean differences, the results revealed that female teachers showed a higher level of global work-related flow experience than male teachers. These findings were consistent with previous research showing that female teachers reported greater engagement and satisfaction with the work and lower burnout (Okpara et al., 2005; Rey et al., 2012). This finding may be related to socially constructed gender roles. More specifically, as discussed in Motro and Ellis (2016), the society has a higher expectation for women to carry out communal roles and display the related traits (e.g., friendliness, sympathy, gentleness, caring, and kindness, etc.). On the other hand, society has a higher expectation for men to carry out agentic roles and display these associated traits (e.g., power, dominance, independence, aggression, and competence, etc.). The theory about role congruity suggests that, when a group’s stereotype is not matched with the expected social roles, biased responses may occur (Diekman and Hirnisey, 2007). Due to the incongruity between the demands of teaching and the typically expected societal roles of males, male teachers may experience lower level of flow.

The relationships between the WOLF factors with external factors of autonomy and job satisfaction supported the nomological validity of the WOLF. The global flow experience was found to be positively associated with autonomy, and this makes sense, as previous research (e.g., Fried and Ferris, 1987; Saavedra and Kwun, 2000) indicated that when employees could schedule their work and determine some aspects of their job, this could contribute to the employees’ positive affect and motivation. This finding is in line with the empirical findings in previous research related to the job characteristics model (Hackman and Oldham, 1980), and to the job demands-resources model (Bakker and Demerouti, 2007), in that high levels of job resources (e.g., autonomy and social support) lead to work-related flow (Zito et al., 2016).

The other finding that the overall work-related flow was positively related to job satisfaction is also in line with previous research, which indicated that flow experience had an important effect on job satisfaction (Geyser et al., 2015), and the psychological state of flow was considered critical in redesigning interventions in the workplace in order to promote job satisfaction (Maeran and Cangiano, 2013). Our results also revealed that only the specific factors of work enjoyment and intrinsic work motivation, but not the absorption S-factor, had positive relationship with job satisfaction and autonomy, confirming the notion that absorption might have some overlap with the holistic description of flow (Bakker, 2008).

Despite the strength of this study in using systematic modeling approaches to examine the latent structure of the WOLF, there are some limitations in this study. One limitation is that the study relied on a convenience sample of Chinese teachers, which may limit the generalizability of findings to a wider context. Future research could use samples from other cultures and from other types of employees. Another limitation is that our assessment of the underlying structure of the WOLF was based on cross-sectional data only. Future research may consider the longitudinal stability of the B-ESEM structure.

In summary, our results supported that the B-ESEM solution could best represent the underlying structure of WOLF scores, and this model incorporates two aspects of psychometric multidimensionality: one is the result of the conceptual adjacency of content domains of flow (e.g., work enjoyment and intrinsic work motivation), and the other is associated with the coexistence of the global work-related flow and the three specific components. Furthermore, the strict gender-group measurement invariance of the B-ESEM model was supported. Female teachers, however, showed a higher level of global work-related flow experience than the male teachers. Finally, the nomological validity of WOLF ratings was supported by the statistical relationships of the WOLF factors with job satisfaction and autonomy.

## Data Availability Statement

The datasets generated for this study are available on request to the corresponding authors.

## Ethics Statement

The study protocol was approved by Hunan Normal University Research Ethnics Committee. All participants gave written informed consent.

## Author Contributions

HG and ZW designed the study. HG and XF wrote the first draft and revised the manuscript.

## Conflict of Interest

The authors declare that the research was conducted in the absence of any commercial or financial relationships that could be construed as a potential conflict of interest.

## References

[B1] Asparouhov T. Muthén B. O. (2009). Exploratory structural equation modeling. *Struct. Equ. Model.* 16 397–438. 10.1080/10705510903008204

[B2] Bãdoiu A. Oprea B. (2018). The work-related flow (WOLF) inventory: Romanian adaptation. *Hum. Resour. Psychol.* 16 94–106.

[B3] Bakker A. B. (2008). The work-related flow inventory: construction and initial validation of the WOLF. *J. Vocat. Behav.* 72 400–414. 10.1016/j.jvb.2007.11.007

[B4] Bakker A. B. Demerouti E. (2007). The job demands-resources model: state of the art. *J. Manag. Psychol.* 22 309–328. 10.3390/ijerph17010069 31861812 PMC6981377

[B5] Brislin R. (1986). “The wording and translation of research instruments,” in *Field Methods in Cross-Cultural Research*, eds Lonner W. J. Berry J. W. (Beverly Hills, CA: Sage), 137–164.

[B6] Chen F. F. (2007). Sensitivity of goodness of fit indices to lack of measurement invariance. *Struct. Equ. Model.* 14 464–504. 10.1590/2237-6089-2017-0093 29768529

[B7] Chen Y. Yu X. Huang B. (2016). “The Chinese version of work-related flow inventory (WOLF): an examination of reliability and validity,” in *Paper Presented at the International Conference on Humanities & Social Science*, London.

[B8] Christensen M. (2009). *Validation and Test of Central Concepts in Positive Work and Organizational Psychology: The Second Report from the Nordic Project Positive Factors at Work.* Copenhagen: Renouf Publishing Company Limited.

[B9] Colombo L. Zito M. Cortese C. G. (2013). The Italian version of the work-related flow inventory (WOLF): first psychometric evaluations. *BPA Appl. Psychol. Bull.* 61 37–42.

[B10] Csikszentmihalyi M. (1975). *Beyond Boredom and Anxiety: Experiencing Flow in Work and Play*, 2nd Edn San Francisco, CA: Jossey Bass.

[B11] Csikszentmihalyi M. (1997). *Finding Flow: The Psychology of Engagement with Everyday Life.* New York, NY: HarperCollins.

[B12] Davis F. D. Bagozzi R. P. Warshaw P. R. (1992). Extrinsic and intrinsic motivation to use computers in the workplace. *J. Appl. Soc. Psychol.* 22 1111–1132.

[B13] Deci E. L. Ryan R. M. (1985). The general causality orientations scale: self-determination in personality. *J. Res. Pers.* 19 109–134. 10.1016/0092-6566(85)90023-6

[B14] Delle Fave A. Massimini F. (2003). Optimal experience in work and leisure among teachers and physicians: individual and bio-cultural implications. *Leisure Stud.* 22 323–342. 10.1080/02614360310001594122

[B15] Demerouti E. Bakker A. B. Sonnentag S. Fullagar C. J. (2012). Work-related flow and energy at work and at home: a study on the role of daily recovery. *J. Organ. Behav.* 33 276–295. 10.1002/job.760

[B16] Diekman A. B. Hirnisey L. (2007). The effect of context on the silver ceiling: a role congruity perspective on prejudiced responses. *Pers. Soc. Psychol. Bull.* 33 1353–1366. 10.1177/0146167207303019 17933733

[B17] Fagerlind A. C. Gustavsson M. Johansson G. Ekberg K. (2013). Experience of work-related flow: does high decision latitude enhance benefits gained from job resources? *J. Vocat. Behav.* 83 161–170. 10.1016/j.jvb.2013.03.010

[B18] Freitas C. P. P. Damásio B. F. Haddad E. J. Koller S. H. (2019). Work-related flow inventory: evidence of validity of the Brazilian version. *Paidéia* 29:e2901.

[B19] Fried Y. Ferris G. R. (1987). The validity of the job characteristics model: a review and meta-analysis. *Pers. Psychol.* 40 287–322. 10.1111/j.1744-6570.1987.tb00605.x

[B20] Fullagar C. Kelloway E. K. (2009). “Flow” at work: an experience sampling approach. *J. Occup. Organ. Psychol.* 81 595–615. 10.1348/096317908x357903

[B21] Geyser I. Geldenhuys M. Crous F. (2015). The dimensionality of the work related flow inventory (WOLF): a South African study. *J. Psychol. Afr.* 25 282–287. 10.1080/14330237.2015.1078084

[B22] Gu H. Wen Z. Fan X. (2015). The impact of wording effect on reliability and validity of the core self-evaluation scale (CSES): a bi-factor perspective. *Pers. Individ. Dif.* 83 142–147. 10.1016/j.paid.2015.04.006

[B23] Gu H. Wen Z. Fan X. (2017a). Examining and controlling for wording effect in a self-report measure: a Monte Carlo simulation study. *Struct. Equ. Model.* 24 545–555. 10.1080/10705511.2017.1286228

[B24] Gu H. Wen Z. Fan X. (2017b). Structural validity of the Machiavellian personality scale: a bifactor exploratory structural equation modeling approach. *Pers. Individ. Dif.* 105 116–123. 10.1016/j.paid.2016.09.042

[B25] Hackman J. R. Oldham G. R. (1980). *Work Redesign.* Reading, MA: Addison- Wesley.

[B26] Happell B. Gaskin C. J. Platania-phung C. (2015). The construct validity of the Work-related flow inventory in a sample of Australian workers. *J. Psychol.* 149 42–62. 10.1080/00223980.2013.838539 25495162

[B27] Hu L. T. Bentler P. M. (1999). Cutoff criteria for fit indexes in covariance structure analysis: conventional criteria versus new alternatives. *Struct. Equ. Model.* 6 1–55. 10.1080/10705519909540118

[B28] Jackson S. A. Marsh H. W. (1996). Development and validation of a scale to measure optimal experience: the flow state scale. *J. Sport Exerc. Psychol.* 18 17–35. 10.1123/jsep.18.1.17

[B29] Lin C. P. Joe S. W. (2012). To share or not to share: assessing knowledge sharing, inter-employee helping, and their antecedents among online knowledge workers. *J. Bus. Ethics* 108 439–449. 10.1007/s10551-011-1100-x

[B30] Llorens S. Salanova M. Rodríguez A. M. (2013). How is flow experienced and by whom? Testing flow among occupations. *Stress Health* 29 125–137. 10.1002/smi.2436 22674654

[B31] Locke E. A. (1976). “The nature and causes of job satisfaction,” in *Handbook of Industrial and Organizational Psychology*, ed. Dunnette M. D. (Chicago, IL: Rand McNally), 1297–1343.

[B32] Maeran R. Cangiano F. (2013). Flow experience and job characteristics: analyzing the role of flow in job satisfaction. *TPM Test. Psychom. Methodol. Appl. Psychol.* 20 13–26.

[B33] Mäkikangas A. Bakker A. B. Aunola K. Demerouti E. (2010). Job resources and flow at work: modeling the relationship via latent growth curve and mixture model methodology. *J. Occup. Health Psychol.* 83 795–814. 10.1348/096317909x476333

[B34] Marsh H. W. Hau K. T. Wen Z. (2004). In search of golden rules: comment on hypothesis testing approaches to setting cutoff values for fit indexes and dangers in overgeneralising Hu & Bentler’s (1999) findings. *Struct. Equ. Model.* 11 320–341. 10.1207/s15328007sem1103_2

[B35] Millsap R. E. (2011). *Statistical Approaches to Measurement Invariance.* New York, NY: Routledge.

[B36] Morgeson F. P. Delaney-Klinger K. Hemingway M. A. (2005). The importance of job autonomy, cognitive ability, and job-related skills for predicting role breadth and job performance. *J. Appl. Psychol.* 90 399–406. 10.1037/0021-9010.90.2.399 15769248

[B37] Morin A. J. S. Arens A. K. Marsh H. W. (2016). A bifactor exploratory structural equation modeling framework for the identification of distinct sources of construct-relevant psychometric multidimensionality. *Struct. Equ. Model.* 23 116–139. 10.1080/10705511.2014.961800

[B38] Morin A. J. S. Boudrias J.-S. Marsh H. W. McInerney D. M. Dagenais-Desmarais V. Madore I. (2017). Complementary variable- and person-centered approaches to the dimensionality of psychometric constructs: application to psychological wellbeing at work. *J. Bus. Psychol.* 32 395–419. 10.1007/s10869-016-9448-7

[B39] Motro D. Ellis A. P. J. (2016). Boys, don’t cry: gender and reactions to negative performance feedback. *J. Appl. Psychol.* 102 227–235. 10.1037/apl0000175 27808525

[B40] Muthén L. K. Muthén B. O. (2012). *Mplus User’s Guide*, 7th Edn Los Angeles, CA: Muthén & Muthén.

[B41] Okpara J. O. Squillace M. Erondu E. A. (2005). Gender differences and job satisfaction: a study of university teachers in the United States. *Women Manag. Rev.* 20 177–190. 10.1108/09649420510591852

[B42] Perreira T. A. Morin A. J. Hebert M. Gillet N. Houle S. A. Berta W. (2018). The short form of the workplace affective commitment multidimensional questionnaire (WACMQ-S): a bifactor-ESEM approach among healthcare professionals. *J. Vocat. Behav.* 106 62–83. 10.1016/j.jvb.2017.12.004

[B43] Reise S. P. (2012). The rediscovery of bifactor measurement models. *Multivariate Behav. Res.* 47 667–696. 10.1080/00273171.2012.715555 24049214 PMC3773879

[B44] Rey L. Extremera N. Pena M. (2012). Burnout and work engagement in teachers: are sex and level taught important? *Ansiedad Estrés* 18 119–129.

[B45] Rodríguez-Sánchez A. M. Schaufeli W. Salanova M. Cifre E. Sonnenschein M. (2011). Enjoyment and absorption: an electronic diary study on daily flow patterns. *Work Stress* 25 75–92. 10.1080/02678373.2011.565619

[B46] Ryan R. M. Deci E. D. (2000). Self-determination theory and the facilitation of intrinsic motivation, social development, and well-being. *Am. Psychol.* 55 68–78. 10.1037/0003-066x.55.1.68 11392867

[B47] Saavedra R. Kwun S. K. (2000). Affective states in job characteristics theory. *J. Organ. Behav.* 2 131–146. 10.1002/(sici)1099-1379(200003)21:2<131::aid-job39>3.0.co;2-q

[B48] Salanova M. Bakker A. B. Llorens S. (2006). Flow at work: evidence for an upward spiral of personal and organizational resources. *J. Happiness Stud.* 7 1–22. 10.1007/s10902-005-8854-8

[B49] Sánchez-Oliva D. Morin A. J. Teixeira P. J. Carraça E. V. Palmeira A. L. Silva M. N. (2017). A bifactor exploratory structural equation modeling representation of the structure of the basic psychological needs at work scale. *J. Vocat. Behav.* 98 173–187. 10.1016/j.jvb.2016.12.001

[B50] Schriesheim C. Tsui A. N. (1980). “Development and validation of a short satisfaction instrument for use in survey feedback interventions,” in *Paper Presented at the Western Academy of Management Meeting*, Salt Lake, UT.

[B51] Sijtsma K. (2009). On the use, the misuse, and the very limited usefulness of Cronbach’s alpha. *Psychometrika* 74 107–120. 10.1007/s11336-008-9101-0 20037639 PMC2792363

[B52] Spreitzer G. M. (1995). Psychological empowerment in the workplace: dimensions, measurement, and validation. *Acad. Manag. J.* 38 1442–1465. 10.5465/256865

[B53] Stenling A. Ivarsson A. Hassmén P. Lindwall M. (2015). Using bifactor exploratory structural equation modeling to examine global and specific factors in measures of sports coaches’ interpersonal styles. *Front. Psychol.* 6:1303. 10.3389/fpsyg.2015.01303 26388808 PMC4555658

[B54] Tóth-Király I. Morin A. J. S. Bõthe B. Orosz G. Rigó A. (2018). Investigating the multidimensionality of need fulfillment: a bifactor exploratory structural equation modeling representation. *Struct. Equ. Model.* 25 267–286. 10.1080/10705511.2017.1374867

[B55] Ullén F. Örjan de Manzano Almeida R. Magnusson P. K. E. Pedersen N. L. (2012). Proneness for psychological flow in everyday life: associations with personality and intelligence. *Pers. Individ. Dif.* 52 167–172. 10.1016/j.paid.2011.10.003

[B56] Zekioğlu A. Tekingündüz S. Sünbül Ö. (2017). The validity and reliability study of Turkish version of work-related flow inventory (WOLF). *Sanitas Magisterium* 3 61–67.

[B57] Zeng C. (2013). Reliability and validity of work-related flow inventory. *Chin. J. Clin. Psychol.* 25 35–38.

[B58] Zito M. Bakker A. B. Colombo L. Cortese C. G. (2015). A two-step study for the Italian adaptation of the work-related flow (WOLF) inventory: the I-WOLF. *Test. Psychom. Methodol. Appl. Psychol.* 22 553–570.

[B59] Zito M. Cortese C. G. Colombo L. (2016). Nurses’ exhaustion: the role of flow at work between job demands and job resources. *J. Nurs. Manag.* 24 E12–E22. 10.1111/jonm.12284 25612156

[B60] Zubair A. Kamal A. (2015). Authentic leadership and creativity: mediating role of work-related flow and psychological capital. *J. Behav. Sci.* 25 150–171.

