# Supplementary material for: Investigating the Multidimensionality of the Work-Related Flow Inventory (WOLF): A Bifactor Exploratory Structural Equation Modeling Framework
Source: Front Psychol. 2020 May 6;11:740. doi: 10.3389/fpsyg.2020.00740 (PMC7218516; doi:10.3389/fpsyg.2020.00740)
Supplement: Supplementary file 1 [file Table_1.DOC]

Appendix

Table S1

The English and Chinese items of the WOLF.

|  | the original English version of  the WOLF | the Chinese version of  the WOLF |
| --- | --- | --- |
| Absorption (AB) | | |
| *a*_1_ | When I am working, I think about nothing else | 工作时, 我从不开小差 |
| *a*_2_ | I get carried away by my work | 工作让我着迷 |
| *a*_3_ | When I am working, I forget everything  else around me | 工作时, 我忘记了周围的一切 |
| *a*_4_ | I am totally immersed in my work | 工作时, 我完全沉浸在其中 |
| Work Enjoyment (WE) | | |
| *w*_1_ | My work gives me a good feeling | 工作给我带来愉悦 |
| *w*_2_ | I do my work with a lot of enjoyment | 我带着一种享受的心情去工作 |
| *w*_3_ | I feel happy during my work | 工作时, 我感到很快乐 |
| *w*_4_ | I feel cheerful when I am working | 工作时, 我感到心情舒畅 |
| *Intrinsic Work Motivation* (IWM) | | |
| *i*_1_ | I would still do this work, even if I received  less pay | 即使报酬少一些, 我也会做这份工作 |
| *i*_2_ | I find that I also want to work in my free time | 我发现自己在业余时间还会想着工作的事情 |
| *i*_3_ | I work because I enjoy it | 我做这份工作是因为我喜欢它 |
| *i*_4_ | When I am working on something, I am doing  it for myself | 当我着手干某项工作时，我是在为自己做 |
| *i*_5_ | I get my motivation from the work itself, and  not from the reward for it | 我的工作动力来自工作本身, 而不是为了报酬 |

Table S2

Standardized Parameter Estimates for the Two-factor CFA (Model 2) and Two-factor ESEM (Model 3) Models.

|  | two-factor CFA | |  | two-factor ESEM | | |
| --- | --- | --- | --- | --- | --- | --- |
|  | λ | δ |  | AB (λ) | WE/IWM (λ) | δ |
| *Absorption* (AB) |  |  |  |  |  |  |
| *a*_1_ | 0.58 | 0.66 |  | **0.33** | 0.27 | 0.31 |
| *a*_2_ | 0.78 | 0.39 |  | **0.72** | *0.15* | 0.68 |
| *a*_3_ | 0.83 | 0.31 |  | **0.72** | *0.13* | 0.65 |
| *a*_4_ | 0.87 | 0.25 |  | **0.82** | *0.05* | 0.74 |
| *Work Enjoyment/ Intrinsic Work Motivation* (WE/IWM) | | | | | | |
| *w*_1_ | 0.85 | 0.28 |  | *–*0.20 | **1.01** | 0.80 |
| *w*_2_ | 0.90 | 0.19 |  | *0.13* | **0.80** | 0.79 |
| *w*_3_ | 0.85 | 0.28 |  | –0.20 | **1.02** | 0.80 |
| *w*_4_ | 0.88 | 0.23 |  | *0.14* | **0.76** | 0.75 |
| *i*_1_ | 0.64 | 0.59 |  | *0.08* | **0.59** | 0.42 |
| *i*_2_ | 0.43 | 0.82 |  | 0.40 | ***0.12*** | 0.24 |
| *i*_3_ | 0.37 | 0.87 |  | 0.28 | ***0.17*** | 0.17 |
| *i*_4_ | 0.59 | 0.65 |  | *–0.08* | **0.66** | 0.37 |
| *i*_5_ | 0.77 | 0.42 |  | 0.37 | **0.49** | 0.61 |

*Note.* Non-significant loadings (*p* > 0.05) are italicized. Target loadings of the ESEM solution are shown in bold.

Table S3

Standardized Factor Loadings for the B-ESEM Model with Two S-factors and One G-factor (Model 5).

|  | GWF (λ) | S-AB (λ) | S-WE/IWM (λ) | δ |
| --- | --- | --- | --- | --- |
| *Absorption* (AB) | | | | |
| *a*_1_ | 0.46 | **0.33** | 0.23 | 0.63 |
| *a*_2_ | 0.81 | **0.21** | –0.17 | 0.28 |
| *a*_3_ | 0.67 | **0.52** | 0.08 | 0.27 |
| *a*_4_ | 0.72 | **0.48** | *–0.03* | 0.24 |
| *Work Enjoyment/Intrinsic Work Motivation* (WE/IWM) | | | | |
| *w*_1_ | 0.72 | *–0.11* | **0.52** | 0.21 |
| *w*_2_ | 0.90 | –0.15 | ***0.21*** | 0.13 |
| *w*_3_ | 0.69 | *–0.06* | **0.59** | 0.17 |
| *w*_4_ | 0.85 | *–0.09* | ***0.23*** | 0.22 |
| *i*_1_ | 0.52 | 0.19 | **0.42** | 0.52 |
| *i*_2_ | 0.50 | *0.06* | ***–0.12*** | 0.74 |
| *i*_3_ | 0.31 | 0.34 | ***0.23*** | 0.74 |
| *i*_4_ | 0.43 | *0.12* | **0.49** | 0.56 |
| *i*_5_ | 0.79 | *0.05* | ***0.08*** | 0.37 |

*Note.* Non-significant loadings (*p* > 0.05) are italicized. Target loadings on specific factors of the B-ESEM solution are shown in bold.
